# Active surveillance in intermediate risk prostate cancer: is it safe?

**DOI:** 10.1590/S1677-5538.IBJU.2016.03.04

**Published:** 2016

**Authors:** Nishanth Krishnananthan, Nathan Lawrentschuk

**Affiliations:** 1 University of Melbourne, Department of Surgery, Austin Health, Melbourne, Australia;; 2 Department of Surgical Oncology, Peter MacCallum Cancer Centre, Melbourne, Victoria, Australia;; 3 Olivia Newton-John Cancer Research Institute, Melbourne, Australia

**Keywords:** Prostatic Neoplasms, Prostate cancer, familial [Supplementary Concept], Watchful Waiting, Disease, Therapeutics

**Opinion: No**

## INTRODUCTION

Active surveillance (AS) is a management strategy for early-stage prostate cancer (PCa) designed to balance early detection of aggressive disease and overtreatment of indolent disease (1). It is advocated as the treatment of choice for favourable-risk disease in several national guidelines (National Comprehensive Cancer Network, National Institute for Health and Clinical Excellence) (2). Despite it’s significant role in low risk PCa, AS is not established as a standard of care for intermediate risk disease. A contemporary registry-based population study in Australia ascertained the treatment trends and patterns of care of 980 men with PCa on AS. It reported that 251 men (8.9%, Median 70.4) with intermediate risk were placed in AS, of whom 53.8% had Gleason score (GS) 3+4 PCa and 10.4% with 4+3 disease (3). The most recent update of the CaPSURE database, a longitudinal, observational study of approximately 15.000 men with all stages of biopsy-proven prostate cancer, also reflected this trend in AS, but questions remain about the safety of this practice and its role in intermediate risk disease.

### Literature and Evidence

Active surveillance consists of close observation via a regimen of periodic PSA measurements, digital rectal examinations and serial prostate biopsies, with the goal of offering curative therapy in the event of disease progression or reclassification (4). Despite long-term data having confirmed the safety and efficacy of AS for low-risk cancers with 10 and 15-year actuarial cause-specific survival rates of 98.1 and 94.3%, respectively (5), the evidence does not extend to completely support its use in intermediate-risk disease. A retrospective analysis of 2.323 patients with localized GS 3+4 prostate cancer who underwent a radical prostatectomy between 2005 and 2013 from 6 academic centres found that 46% of patients with biopsy GS 3+4 cancer have unfavourable disease at final pathology (6). When applying the University of Toronto, Royal Marsden Hospital and Prostate Cancer Research International Active Surveillance (PRIAS) criteria (7) to the above cohort, 78, 59 and 20% of men were eligible for AS, respectively, and the risks of unfavourable disease were decreased to only 42.4, 41.0 and 30.5%, respectively (8). The Cancer Council Ontario (CCO) currently recommends active treatment (surgery or radiotherapy) for patients with intermediate-risk localized prostate cancer (9).

A number of studies also support the role of surgery in men in this intermediate risk group. The PIVOT study found men with intermediate-risk tumours who underwent radical prostatectomy (PSA 10.1 to 20.0ng/ml, GS 7, or a stage T2b tumour) had a 31% relative reduction in all-cause mortality, as compared with those assigned to observation (HR 0.69; 95% CI, 0.49 to 0.98; ARR 12.6%) (10). PCa mortality in this group was not significant despite a similar trend. This was compared with the Scandinavian Prostate Cancer Group 4 (SPCG-4) trial of radical prostatectomy versus watchful waiting in men with prostate cancer, which showed the benefit of surgery in relation to death from PCa was largest in those with intermediate-risk prostate cancer (relative risk, 0.38) (11). There was a significant absolute reduction in men with intermediate risk disease in overall mortality, rate of death from PCa, and in the risk of metastases (11%).

Klotz et al. performed a large cohort Canadian study with 993 patients and up to 16 years of follow-up, with 25% of the patients fulfilling the D’Amico criteria for intermediate risk (5). There were 15 deaths (2.8%) due to PCa in total, all of whom had confirmed metastases before death. 12 (44%) of the 28 patients with metastases had a Gleason score of 3+4 at diagnosis; with a median time to metastasis was 7.3 years (95% CI, 5.81 to 8.76 years). Only 2 of the 28 patients who developed metastasis were not upgraded to GS 7 before developing metastatic disease, neither of whom had surgical grading. Klotz et al. therefore suggested that in a screened population, only selected men older than age 70 years with intermediate-risk PCa are candidates for surveillance (the 15-year PCa mortality is low). This was consistent with Cooperberg et al. who followed 640 men on AS at University of California, San Francisco (UCSF). Among 74 men on AS electing to undergo RP, 16 had intermediate risk disease, with 50% of these patients having pT3 disease (P=09). AS was therefore recommended only for low volume GS 3+4 patients, particularly those with comorbid conditions and appropriate counselling prior to AS. The American Society of Clinical Oncology (ASCO) has recently reinforced the CCO guidelines and also recommended AS for only select patients with low-volume, intermediate-risk patients, with factors such as younger age, prostate cancer volume, patient preference, and ethnicity taken into account when making a management decisions (12).

## DISCUSSION

Although the lifetime risk of receiving a diagnosis of prostate cancer is about 17%, the risk of dying from the disease is approximately 3%, suggesting that conservative management may be appropriate for select men (13). Thus, the way forward for AS must be lighted by improved tools for risk stratification at diagnosis and for early identification of progressive disease (14). A recent systematic review of novel tools for improving patient selection and monitoring low-risk prostate cancer by AS found that magnetic resonance imaging (MRI) has a high specificity for low-risk prostate cancer and new serum markers are associated with unfavourable disease (15). The potential of multi-parametric MRI lies in its high negative predictive value (80-90%) for the intermediate endpoint of disease upgrading, which may make it useful as an AS endpoint predictor (16). It is also beneficial in identifying anterior and higher volume tumours, as well as aiding in disease reclassification. A significant proportion of low risk patients do harbour more aggressive disease, and MRI can provide better risk stratification in both low and intermediate risk patients. This observation is demonstrated by several series of patients meeting AS criteria who underwent radical prostatectomy, revealing Gleason score upgrading in 23%-56% (17). Klotz et al. observed that 16% of patients had histological progression (2). Such observations encourage the need for future research of both MRI and molecular biomarkers such as PCA3 or PSA isoforms in these patients.

When managed with non-curative intent, intermediate-risk PCa is associated with 10-year and 15-year prostate-cancer-specific mortality rates of 13 and 19.6% (18). Thus, men with this disease are at risk of developing incurable disease in the future as they may miss the window of curability when opting for AS (8). Thomsen et al. estimated average 5-year and 10-year probabilities of discontinuing AS at 33% (14%–41%) and 55% (40%–59%) respectively, with a majority undergoing delayed curative treatment (RP or RT) (19). Thus, AS can only be warranted in select intermediate risk PCa patients, with consideration for individual tumour metrics, patient age and overall health, as well as patient preferences and the potential side effects of curative treatments. For those young patients (<65) with longer quality-adjusted life expectancy in this group, surgery should still be considered the definitive approach.

## CONCLUSIONS

At least 50%-60% of individuals diagnosed with PCa ultimately die of other causes, and as a result, AS has become a chosen management option for low risk PCa patients (20). The latest literature however, demonstrates surgery as the mainstay of treatment in intermediate risk patients, with the role of AS limited to select men in this group. The use of MRI and prostate serum and genetic markers are still being evaluated, and until that time, it is recommended that definitive intervention remain the optimal choice of management in this group of patients.
